# Tetraspanin CD9 Is a Positive Regulator of Filovirus Egress

**DOI:** 10.3390/v18010104

**Published:** 2026-01-13

**Authors:** Loveleena K. Anand, Marija A. Djurkovic, Ariel Shepley-McTaggart, Olena Shtanko, Ronald N. Harty

**Affiliations:** 1 Department of Pathobiology, School of Veterinary Medicine, University of Pennsylvania, 3800 Spruce Street, Philadelphia, PA 19104, USA; 2 Integrated Biomedical Science Program, University of Texas San Antonio, San Antonio, TX 78227, USA; 3 Host-Pathogen Interactions, Texas Biomedical Research Institute, 8715 W. Military Drive, San Antonio, TX 78227, USA

**Keywords:** Ebola, Marburg, filovirus, tetraspanin CD9, VLP egress, virus–host interaction

## Abstract

Filoviruses, including Ebola (EBOV) and Marburg (MARV) viruses, are zoonotic pathogens that cause severe hemorrhagic fever in humans, with mortality rates reaching up to 90%. Filovirus egress and spread are driven by the viral matrix protein VP40 and regulated both positively and negatively by a growing number of specific host interactors. Here, we identify tetraspanin protein CD9, a plasma membrane organizing and scaffolding protein, as playing a role in facilitating efficient egress of EBOV and MARV. Indeed, we observed a significant decrease in viral egress of VLPs and live filoviruses from CD9-KD cells as compared to that from WT cells. Moreover, exogenous expression of CD9 rescued egress of VP40 VLPs close to WT levels in the CD9-KD cells. These findings identify tetraspanin CD9 as a positive regulator of filovirus egress, and thus CD9 may represent a potential new target for antiviral therapies targeting the late stage of the filovirus lifecycle.

## 1. Introduction

EBOV and MARV are global public health threats that can cause both acute hemorrhagic fever and chronic persistent infections in humans [1,2]. Notably, these viruses can cross cellular barriers/junctions to establish persistent infections in immunologically privileged sites (e.g., CNS) typically inaccessible to antibody therapy, resulting in longer term pathologic consequences [3,4,5,6]. It is clear that host proteins play critical roles in regulating the filovirus lifecycle, and thus a better understanding of the interplay between the virus and host will allow for the identification of new therapeutic targets and the development of new countermeasures that could be used to target both acute and persistent infections [7,8].

The plasma membrane and its associated protein networks play essential and tightly coordinated roles in regulating filovirus entry, assembly, and egress. Increasing evidence indicates that viruses such as EBOV and MARV exploit membrane organization, cytoskeletal architecture, and extracellular matrix (ECM) interactions to complete their lifecycle. For example, we recently identified the filamin family of actin crosslinking proteins as playing a key role in regulating entry of filoviruses via macropinocytosis [9]. Notably, the filamin proteins function as scaffolding proteins to coordinate the assembly of membrane complexes and their interactions with the extracellular matrix (ECM) to dynamically regulate cell signalling, cell adhesion, and motility [10,11]. To explore these pathways, we performed RNA Seq. analysis to identify differentially expressed genes (DEGs) that may contribute to the robust EBOV/MARV phenotypes observed in the filamin A or B knockdown (KD) cells. Of the many DEGs identified, we were particularly intrigued by five genes, each of which were highly differentially expressed and linked to filamin activity, ECM composition, membrane stiffness, membrane microdomain formation, and/or cytoskeletal dynamics as components of the matrisome. These genes include (i) plastin-3 (PLS3), an F-actin-binding/bundling protein that regulates actin dynamics and participates in cell migration, focal adhesions, and cytoskeletal shaping [12,13]; (ii) α-parvin (PARVA), involved in mechanotransduction, regulating cytoskeletal dynamics, cell invasion/migration, ECM adhesions and cell stiffness [14,15,16]; (iii) CarboHydrate SulfoTransferase 15 (CHST15), a Golgi localized type II transmembrane glycoprotein involved in the biosynthesis of highly sulfated disaccharide units of chondroitin sulfates (CSs), critical structural components of the ECM that function in its remodelling during EMT, proliferation, and adhesion [17,18,19]; (iv) podoplanin (PDPN), which helps connect cells to the ECM, promotes epithelial–mesenchymal transition (EMT), and mediates cell migration/invasion and adhesion [20,21,22,23,24]; and (v) tetraspanin CD9, a plasma membrane-associated tetraspanin that serves as a scaffold for forming tetraspanin-enriched microdomains (TEMs) that include many ECM-related proteins [25]. Among these candidates, CD9 stood out due to its well-documented involvement in viral infection processes. CD9 has been implicated in the pathogenesis and regulated entry and/or egress of a diverse range of viruses—including Zika, HIV-1, coronaviruses, and influenza A virus—highlighting its capacity to modulate multiple stages of viral lifecycles through its effects on membrane organization, receptor clustering, and vesicle trafficking [25,26,27,28,29,30,31,32]. This strong intersection between CD9 function, ECM-associated pathways, and viral exploitation positions CD9 as a particularly compelling host factor for further investigation in the context of EBOV and MARV infection.

Here, we focused on the late stage of filovirus egress and sought to determine whether expression of CD9 affected egress of EBOV and MARV VLPs from Hela cells, and whether CD9 affected infectivity and egress of authentic EBOV and MARV in Hela cells. We generated CD9-KD cells using CRISPR/Cas9 technology as well as siRNA approaches, and asked whether reduced levels of endogenous CD9 in the KD cells modulated infectivity of live EBOV and MARV and egress of both VP40 VLPs and live EBOV and MARV. Our results showed that egress of VLPs and live virus was reduced significantly in the CD9-KD cells as compared to that in WT cells. Importantly, we were able to restore egress of EBOV and MARV VLPs from the CD9-KD cells back to WT levels by transiently expressing exogenous CD9. Overall, our findings identify CD9 for the first time as a positive regulator of filovirus egress, positioning it as a promising host candidate for antiviral strategies aimed at the late budding stage of the filovirus lifecycle.

## 2. Materials and Methods

### 2.1. Cell Lines and Reagents

Human cervical carcinoma (Hela) cell line, HT-1080 cells, FLNA-KD, FLNB-KD, CD9-WT, and CD9-KD cells were maintained as monolayers in a humidified 5% CO_2_ incubator at 37 °C. The cultures were maintained in DMEM (Corning; Glendale, AZ, USA), supplemented with 10% FBS (Gibco; Waltham, MA, USA), and penicillin (100 U/mL)/streptomycin (100 μg/mL) (Invitrogen; Carlsbad, CA, USA).

### 2.2. Bulk RNA Sequencing

WT HT-1080, FLNA-KD, and FLNB-KD cells were lysed in Trizol Reagent (Invitrogen) and total RNA was extracted using the RNeasy Plus Micro Kit (Qiagen; Germantown, MD, USA) following the manufacturer’s instructions. Total RNA was examined for quantity and quality before Bulk RNA Sequencing. Raw reads were mapped to the human reference transcriptome using Kallisto, version 0.46.2.

### 2.3. CRISPR/Cas9 Assay

Hela CD9-KD cells were generated by CRISPR-cas9-mediated knockout system. Parent Hela Cells Cells (2.5 × 10^5^ cells/transfection) were propagated in 3 mL of antibiotic-free growth media, 24 h prior to transfection. Healthy cultures were transfected with “X-tremeGENE HP DNA Transfection Reagent”, CRISPR/cas9 KD plasmid (sc-400252), and HDR plasmid (sc-400252), following the manufacturer’s instructions. The clones were subsequently selected for puromycin susceptibility and validated as KD by Western blotting.

### 2.4. siRNA Assay

Hela cells (2.5 × 10^5^ cells/transfection) were seeded in DMEM along with 10% FBS prior to transfection. Subsequently, cells were maintained in Opti-MEM (Gibco, 31985-070) and transfected twice with either control (random) or CD9-specific siRNAs (Santa Cruz Biotechnology; Dallas, TX, USA) along with VP40 using Lipofectamine (Invitrogen, 11668027). Cells and supernatants were harvested at the indicated times post-transfection and the designated proteins were detected by Western blotting.

### 2.5. Plasmids

The Flag-tagged mVP40-WT and untagged eVP40 plasmids were described previously [33]. CD9-optimized CRISPR/Cas9 KD plasmid (h), HDR Plasmid (sc-400252), and CD9 CRISPR Activation Plasmid (h) were purchased from Santa Cruz Biotechnology (sc-400252-ACT). The pCAG-HiBiT-CD9 (162589) was a gift from Masaharu Somiya (Osaka University, Osaka, Japan) (Addgene plasmid # 162589). For transfections, plasmid DNAs were incubated with Lipofectamine^TM^ 2000 (Invitrogen; Carlsbad, CA, USA) at 1:10 DNA:Lipofectamine mass ratio, in Opti-MEM (Gibco; Waltham, MA, USA) for 20 min at room temperature (RT), then added gradually to adherent cell cultures.

### 2.6. Viruses

All work with replication-competent viruses was performed at Texas Biomedical Research Institute (San Antonio, TX, USA) according to approved standard operating procedures and protocols approved by the Institute’s Biohazard and Safety and Recombinant DNA Committees. The NCBI database accession numbers for virus stocks used in this study were KF990213 (the recombinant EBOV variant Mayinga encoding GFP) and NC_001608 (Marburg virus strain Musoke) [34,35]. The viral stocks were amplified in Vero cells in DMEM supplemented with 2% FBS for 7 days. The culture supernatants were clarified of cell debris, then overlaid over a 20% sucrose cushion in PBS, and ultracentrifuged at 28,000 rpm for 2 h at 4 °C. Pelleted virus resuspended in PBS was titrated by using conventional plaque assays.

### 2.7. VP40 VLP Budding Assay

In brief, WT and KD cells were transfected with identical quantities of the specified plasmids (eVP40 and mVP40) using Lipofectamine (Invitrogen). The following day, the cell culture medium was collected and clarified at 2500 rpm for 10 min, then layered onto a 20% sucrose cushion in STE buffer (0.01 M Tris-HCl [pH 7.5], 0.01 M NaCl, 0.001 M EDTA [pH 8.0]) and centrifuged at 36,000 rpm for 2 h at 4 °C. Pelleted VLPs were resuspended in 40 μL of STE buffer overnight at 4 °C and analyzed using SDS-polyacrylamide gel electrophoresis (PAGE) and quantification was performed employing Image J software version 1.54r.

### 2.8. Western Blotting

Cells were solubilized with RIPA buffer 50 mM Tris HCL-pH 7.4 (Sigma: St. Louis, MO, USA, 1185-53-1), 150 Mm NaCl (Sigma: 31434), and 1%Triton X-100 (Sigma: L4509) along with 1% protease Cocktail inhibitor (Sigma: P8215) and Phosphatase inhibitor (Sigma: S42629). Lysates were analyzed using SDS PAGE buffer, and proteins were detected using Blue-sensitive X-ray films and the Immobilon Western chemiluminescent HRP substrate (Millipore: WBKLSO500; Burlington, MA, USA).

### 2.9. Antibodies

The primary antibodies used in the study include mouse CD9 monoclonal antibody (ProteinTech; Rosemont, IL, USA, cat no-60232-1), mouse beta-actin antibody (GeneTex; Irvine, CA, USA, cat no-GTX629630), mouse anti-FLAG antibody (ProteinTech, 66008-4-Ig) to detect FLAG-tagged mVP40, and rabbit anti-eVP40 polyclonal antibody (IBT Services; Leesburg, VA, USA, cat no 0301-010). The secondary antibodies used in this study include mouse IgG HRP-linked whole Ab (NA931, Millipore) to detect the primary anti-FLAG mVP40 antibody, and rabbit IgG HRP-linked whole Ab (NA934, Millipore) to detect the primary eVP40 antibody.

### 2.10. Statistics and Reproducibility

Statistical analysis was performed with Graph Pad Prism 10.4.1 for Windows. Details of statistical analyses performed using data from at least three independent experiments are stated in the figure legends. The one-way/two-way ANOVA analyses and two-tailed unpaired *t*-test were used as indicated in the figure legends.

## 3. Results

### 3.1. RNA Seq. Analysis Identifies CD9 as a Highly Differentiated Gene in WT vs. Filamin A or B Knockdown Cells

**Figure 1:**
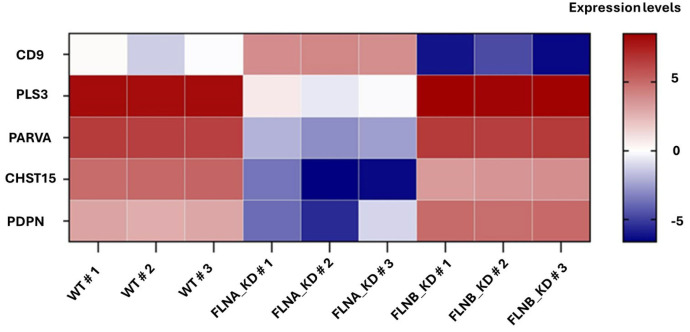
Heat map showing differential expression and fold change of genes CD9, PLS3, PARVA, CHST15, and PDPN in 3 biological replicates of WT HT-1080, FLNA-KD, and FLNB-KD cells.

CD9 was identified by RNA Seq. analysis of three biological samples as a highly differentially expressed gene (DEG) in WT vs. Filamin A or B knockdown cells (Figure 1), and we speculated that CD9 may contribute to the robust EBOV/MARV phenotypes observed in the filamin A or B knockdown (KD) cells [9]. Of the many DEGs identified, we highlight a subset of these data that include CD9 and four additional DEGs, each of which are linked to filamin activity, ECM composition, membrane stiffness, membrane microdomain formation, and/or cytoskeletal dynamics as components of the matrisome (Figure 1). Since CD9 has been shown to play a role in the entry and egress stages of a wide array of viruses, we focused our efforts on CD9 to determine whether it plays a role in the late budding stage of EBOV and MARV.

### 3.2. Knockdown of Endogenous CD9 Inhibits Egress of EBOV and MARV VP40 VLPs

**Figure 2:**
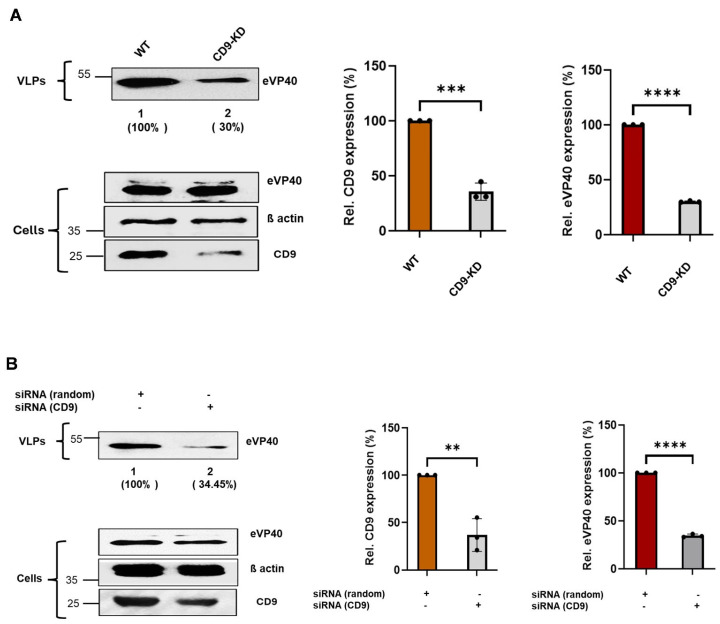
Knockdown of endogenous CD9 inhibits egress of eVP40 VLPs. (**A**) Representative Western blot showing the indicated proteins in cell lysates and VLPs from WT and CRISPR/Cas9-generated CD9-KD cells, and bar graphs depicting the quantification of CD9 in cell extracts (left graph) and eVP40 expression levels in VLPs from WT and CD9-KD cells (right graph) from 3 independent experiments. (**B**) Representative Western blot showing the indicated proteins in cell lysates and VLPs from random siRNA and CD9-specific siRNA treated cells. Bar graphs represent quantification of CD9 in cell extracts (left graph) and eVP40 expression levels in VLPs from WT and CD9-KD cells (right graph) from 3 independent experiments. Mean ± SD is shown (*n* = 3), and statistical analysis was performed using an unpaired *t*-test (**** *p* < 0.0001, *** *p* = 0.0001, ** *p* < 0.0032). Antibodies used to detect the indicated proteins are described in the Materials and Methods. Percentages shown in () indicate the percent level of eVP40 in VLPs compared to the WT control set at 100%.

Here, we sought to determine whether knockdown of endogenous CD9 expression would affect egress of EBOV VP40 VLPs. We used CRISPR/Cas9 technology to generate a stable Hela cell line in which endogenous expression of CD9 was knocked down (CD9-KD), and we used siRNA knockdown as a second complementary approach to determine whether expression of endogenous CD9 is required for efficient egress of EBOV VP40 VLPs (Figure 2). Briefly, WT and CD9-KD Hela cells were transfected with an eVP40 expression plasmid, and eVP40 protein levels were detected and quantified in both cell lysates and VLPs at 24 h post-transfection using our well-established VP40 VLP budding assay (Figure 2A). As expected, expression levels of endogenous CD9 were reduced significantly in CD9-KD cell lysates as compared to those in WT lysates, whereas levels of eVP40 and β-actin in the CD9-KD and WT lysates were equivalent (Figure 2A; Cells). Notably, egress of eVP40 VLPs was reduced significantly in the CD9-KD cells as compared to that from WT Hela cells in multiple independent experiments (Figure 2A). Similar findings were observed using an siRNA approach (Figure 2B). Indeed, egress of eVP40 VLPs was reduced in cells treated with CD9-specific siRNAs compared to that in cells treated with non-specific random siRNA controls (Figure 2B). These results suggest that expression of endogenous CD9 in Hela cells is required for efficient egress of EBOV VP40 VLPs.

**Figure 3:**
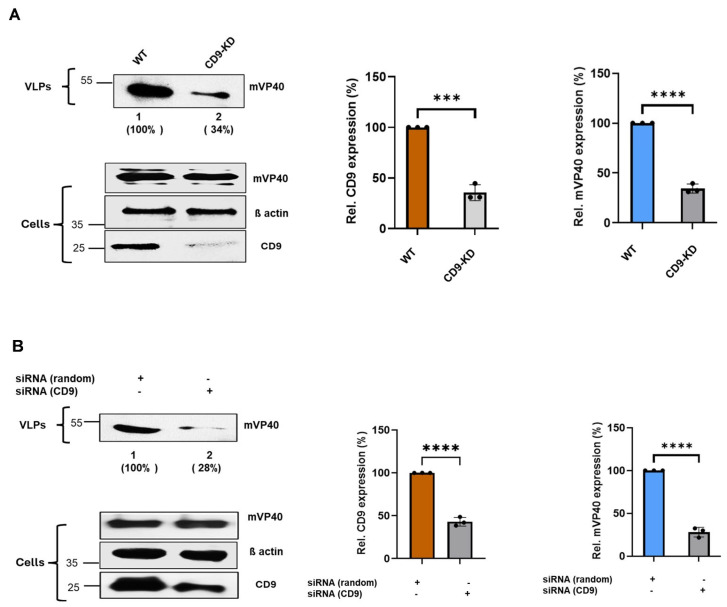
Knockdown of endogenous CD9 inhibits egress of mVP40 VLPs. (**A**) Representative Western blot showing the indicated proteins in cell lysates and VLPs from WT and CRISPR/Cas9-generated CD9-KD cells, and bar graphs depicting the quantification of CD9 from cell extracts (left graph) and mVP40 expression levels in VLPs from WT and CD9-KD cells (right graph) from 3 independent experiments. (**B**) Representative Western blot showing the indicated proteins in cell lysates and VLPs from random siRNA and CD9-specific siRNA treated cells. Bar graphs represent quantification of CD9 from cell extracts (left graph) and mVP40 expression levels in VLPs from WT and CD9-KD cells (right graph) from 3 independent experiments. Mean ± SD is shown (*n* = 3), and statistical analysis was performed using an unpaired *t*-test (**** *p* < 0.0001, *** *p* = 0.0001). Antibodies used to detect the indicated proteins are described in the Materials and Methods. Percentages shown in () indicate the percent level of mVP40 in VLPs compared to the WT control set at 100%.

Next, we sought to determine whether knockdown of endogenous CD9 expression would similarly affect egress of MARV VP40 VLPs as it did for EBOV VP40 VLPs. We used the same CRISPR/Cas9 and siRNA approaches described above to determine whether expression of endogenous CD9 is required for efficient egress of MARV VP40 VLPs (Figure 3). WT and CD9-KD Hela cells were transfected with an mVP40 expression plasmid, and mVP40 protein levels were detected and quantified in both cell lysates and VLPs at 24 h post-transfection (Figure 3A). As observed above for eVP40, we observed that egress of mVP40 VLPs was also reduced significantly in the CD9-KD cells, using either CRISPR/Cas9 or siRNA, as compared to that from WT Hela cells in multiple independent experiments (Figure 3A,B). Taken together, these results indicate that expression of endogenous CD9 in Hela cells is required for efficient egress of both EBOV and MARV VP40 VLPs, and thus functions as a positive regulator of filovirus VLP egress.

### 3.3. Exogenous Expression of CD9 Rescues Egress of EBOV and MARV VP40 VLPs in CD9-siRNA Knockdown Cells

**Figure 4:**
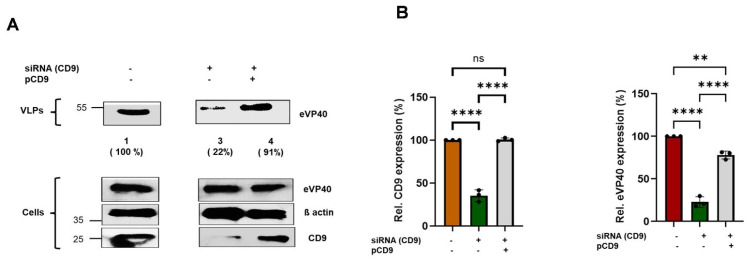
Exogenous expression of CD9 restores eVP40 VLP egress in CD9-siRNA knockdown cells. (**A**) Representative Western blot showing the indicated proteins in cell lysates and VLPs from eVP40 transfected cells (lane 1), eVP40 + CD9-siRNA transfected cells (lane 3), and eVP40 + CD9-siRNA + pCD9 transfected cells (lane 4). (**B**) Bar graphs showing quantification of CD9 in cell extracts (left graph) and eVP40 expression levels in VLPs (right graph) using Image J software. Statistical analysis was performed using one-way ANOVA Bonferroni multiple comparison test (ns = not significant, ** *p* < 0.0024, **** *p* < 0.0001). Antibodies used to detect the indicated proteins are described in the Materials and Methods. Percentages shown in () indicate the percent level of eVP40 in VLPs compared to the WT control set at 100%.

To show more definitively that knockdown of endogenous CD9 is the cause of the budding defect for eVP40 VLPs shown in Figure 2, we asked whether transient expression of exogenous CD9 would rescue the reduced egress of eVP40 VLPs observed in the CD9-siRNA knockdown cells back to WT levels. Briefly, Hela cells were transfected with either eVP40 alone, eVP40 + CD9-siRNA, or eVP40 + CD9 siRNA + a CD9 expression plasmid (pCD9) (Figure 4). Cell lysates and VLPs were harvested, and eVP40 was detected and quantified from cell lysates and purified VLPs (Figure 4). As expected, egress of eVP40 VLPs (Figure 4A) was robust in eVP40 transfected cells (Figure 4A, lane 1) but was reduced significantly in cells treated with CD9-siRNA (lane 2). Notably, we observed that egress of eVP40 VLPs was close to WT levels in cells receiving CD9-siRNA + exogenous pCD9 (lane 3). These results were found in multiple independent experiments (Figure 4B, right panel). Endogenous CD9 was efficiently knocked down in the siRNA-treated cells (compare lanes 1 and 2), and CD9 levels were restored back to WT levels in cells receiving the CD9 expression plasmid (Figure 4A, compare lanes 1 and 3; Figure 4B, left panel).

**Figure 5:**
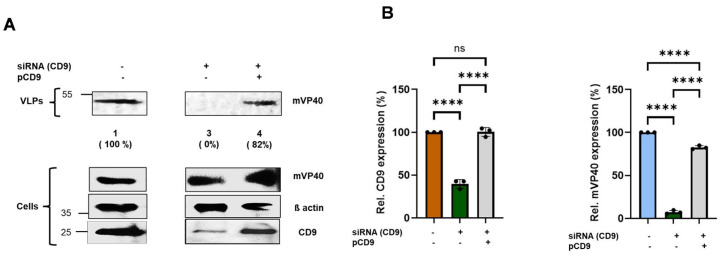
Exogenous expression of CD9 restores mVP40 VLP egress in CD9-siRNA knockdown cells. (**A**) Representative Western blot showing the indicated proteins in cell lysates and VLPs from mVP40 transfected cells (lane 1), mVP40 + CD9-siRNA transfected cells (lane 3), and mVP40 + CD9-siRNA + pCD9 transfected cells (lane 4). (**B**) Bar graphs showing quantification of CD9 in cell extracts (left graph) and mVP40 expression levels in VLPs (right graph) using Image J software. Statistical analysis was performed using one-way ANOVA Bonferroni multiple comparison test (ns = not significant, **** *p* < 0.0001). Antibodies used to detect the indicated proteins are described in the Materials and Methods. Percentages shown in () indicate the percent level of mVP40 in VLPs compared to the WT control set at 100%.

Next, we sought to determine whether exogenous CD9 would also rescue egress of mVP40 VLPs. As described above for eVP40, Hela cells were transfected with either mVP40 alone, mVP40 + CD9-siRNA, or mVP40 + CD9 siRNA + pCD9 (Figure 5). Cell lysates and VLPs were harvested, and mVP40 was detected and quantified from cell lysates and purified VLPs (Figure 5). As expected, egress of mVP40 VLPs (Figure 5A) was efficient in mVP40 transfected cells (Figure 5A, lane 1) but was reduced significantly in cells treated with CD9-siRNA (lane 2). As observed for eVP40 VLPs, we also found that egress of mVP40 VLPs was close to WT levels in cells receiving CD9-siRNA + exogenous pCD9 (lane 3). These results were found in multiple independent experiments (Figure 5B). Taken together, these results strongly indicate that the reduction in eVP40 and mVP40 VLP egress in CD9-siRNA treated cells was indeed due to specific knockdown of endogenous CD9, and not due to off-target effects. These findings support our conclusion that CD9 is a positive regulator of filovirus VLP egress.

### 3.4. Endogenous CD9 Expression Is Required for Efficient Infectivity and Egress of Authentic EBOV and MARV in Hela Cells

**Figure 6:**
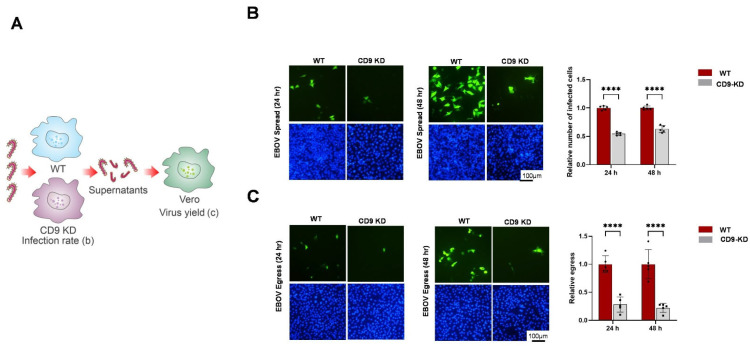
Knockdown of endogenous CD9 inhibits infectivity and egress of live EBOV in Hela cells. (**A**) Schematic diagram showing the EBOV infection protocol in WT and CD9-KD cells. The infection rate or spread in WT and CD9-KD cells is shown in (**B**). Virus-containing supernatants harvested from WT and CD9-KD cells were used to infect fresh monolayers of Vero cells to assess virus yield shown in (**C**). This identical protocol was used for MARV infection shown in Figure 7. (**B**) Representative images showing infectivity and spread of live EBOV-GFP (green) in WT and CD9-KD Hela cells at 24 and 48 h post-infection. Cell nuclei were marked with Hoechst stain (blue). Bar graphs show the quantification of EBOV-GFP-infected cells in quintuplicate. Infection efficiency or virus spread is equal to the number of GFP-positive cells/number of nuclei. (**C**) Supernatants were collected at 24 and 48 h post-infection from EBOV-GFP-infected WT and CD9-KD cells with comparable infection efficiencies. Supernatants were then used to infect fresh monolayers of Vero cells to quantify EBOV-GFP egress. Representative images of infected Vero cells are shown for the 24 and 48 h post-infection time points. Cell nuclei were marked with Hoechst stain (blue). Quantification of virus egress (number of GFP-positive Vero cells) from quintuplicate experiments is shown in the bar graphs. Statistical analysis was performed using a two-way ANOVA with Bonferroni multiple comparison test (**** *p* < 0.0001).

We next sought to determine whether expression of endogenous CD9 was critical for live EBOV infectivity and egress. A schematic diagram of the EBOV infection protocol to assess infectivity and virus yield (egress) is shown (Figure 6A). Wild-type or CD9-KD Hela cells were infected with EBOV-GFP at an MOI of 0.1 in quintuplicate for 24 or 48 h (Figure 6B). To quantify viral egress, a separate set of WT and CD9-KD cells was incubated with EBOV-GFP at different concentrations to identify a condition with a comparable infection efficiency, determined as the ratio of infected cells and nuclei. The supernatants collected from these cells were titrated onto Vero cells for 24 or 48 h (Figure 6C). EBOV-GFP infectivity, or virus spread, was determined as the number of GFP-positive cells divided by the number of nuclei (Figure 6B). We observed a significant decrease in EBOV-GFP infectivity/spread at both 24 and 48 h post-infection in the CD9-KD cells as compared to that in WT Hela cells (Figure 6B). Similarly, virus yield from the CD9-KD cells was reduced significantly (75–80%) as compared to that from the WT cells at 24 and 48 h post-infection (Figure 6C). These findings correlated well with data from our BSL-2 VLP budding assays and further demonstrate that endogenous expression of host CD9 is critical for efficient infectivity/spread and egress of live EBOV in cell culture.

**Figure 7:**
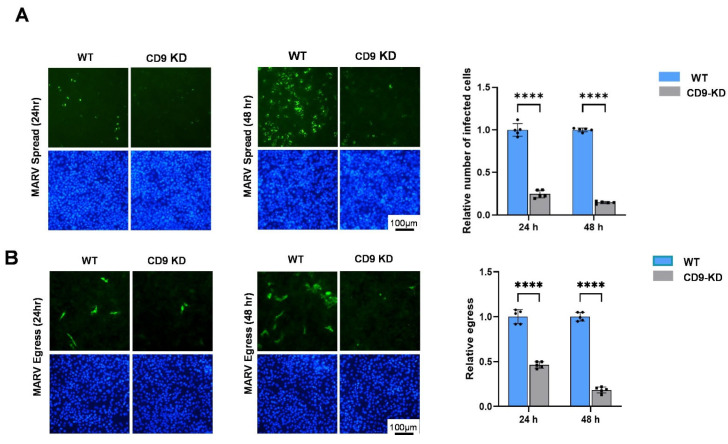
Knockdown of endogenous CD9 inhibits infectivity and egress of live MARV in Hela cells. (**A**) Representative images showing infectivity and spread of live MARV (green) in WT and CD9-KD Hela cells at 24 and 48 h post-infection. MARV-infected cells were stained with antisera against MARV VLPs or NP. Cell nuclei were marked with Hoechst stain (blue). Bar graphs show the quantification of MARV-infected cells in quintuplicate. Infection efficiency or virus spread is equal to the number of infected (green) cells/number of nuclei. (**B**) Supernatants were collected at 24 and 48 h post-infection from MARV-infected WT and CD9-KD cells with comparable infection efficiencies. Supernatants were then used to infect fresh monolayers of Vero cells to quantify MARV egress. Representative images of infected Vero cells are shown for the 24 and 48 h post-infection time points. Cell nuclei were marked with Hoechst stain (blue). Quantification of virus egress (number of MARV-infected [green] Vero cells) from quintuplicate experiments is shown in the bar graphs. Statistical analysis was performed using a two-way ANOVA with Bonferroni multiple comparison test (**** *p* < 0.0001).

Next, we sought to determine whether expression of endogenous CD9 would similarly affect infectivity/spread and egress of live MARV. WT and CD9-KD cells were infected in quintuplicate with MARV at an MOI of 0.1. Cells were imaged and supernatants were collected at 24 and 48 h post-infection and used to infect fresh monolayers of Vero cells. MARV-positive cells were identified using antisera against MARV VLPs or against MARV NP, and infectivity/spread was quantified as the ratio of MARV-positive cells to total cell nuclei (Figure 7A). As with EBOV, we observed a significant reduction in MARV infectivity/spread at both 24 and 48 h post-infection in the CD9-KD cells as compared to that in the WT cells (Figure 7A). In addition, MARV egress was also reduced substantially (up to 80% at 48 h p.i.) in the CD9-KD cells as compared to WT cells (Figure 7B). Taken together, these data demonstrate that expression of endogenous CD9 in Hela cells is crucial for efficient infectivity/spread and egress of two related filoviruses, and that CD9 should be considered as a positive host regulator of filovirus egress.

## 4. Discussion

In this report, we sought to define the role of CD9 in regulating the plasma membrane-mediated stage of filovirus egress. CD9 emerged as one of the most highly differentially expressed genes in our RNA-Seq analysis comparing WT cells to filamin A/B knockdown cells. Notably, several additional DEGs (PLS3, PARVA, CHST15, and PDPN) were also detected and belong to functionally related classes within the matrisome (Figure 1). The enrichment of ECM-associated genes among the top DEGs suggests that filovirus egress may not rely on a single host factor, but rather on a broader ECM-regulated framework that shapes plasma membrane mechanics and cytoskeletal organization. This pattern further reinforces the idea that the ECM and its associated proteins could play previously unappreciated roles in governing EBOV and MARV release. These findings prompted us to investigate CD9 as a potential regulator of filovirus egress, reasoning that it may act in concert with filamin and other ECM-associated matrisome proteins to influence this plasma-membrane-driven stage of the filovirus lifecycle.

CD9 is a member of the tetraspanin family of cell surface proteins and is characterized by four transmembrane domains [36]. It plays a key role in multiple cellular processes, including regulating cell adhesion, migration, and signal transduction by organizing membrane microdomains, as well as interacting with integrins and other cell surface receptors [37,38,39]. Expression of CD9 has been shown to influence viral infection processes through its role in organizing membrane microdomains and facilitating interactions with host and viral proteins. Indeed, several viruses were shown to exploit CD9 to enhance their entry (infectivity), replication, and/or spread. For example, CD9 can modulate the fusion of viral and host cell membranes, impacting infections by viruses such as HIV, hepatitis C virus (HCV), and human papillomavirus (HPV) [25,40,41,42]. CD9 has also been linked to the formation of extracellular vesicles and exosomes, which some viruses usurp for intercellular transmission. Indeed, CD9 plays an important role in virus egress by regulating membrane organization and vesicle trafficking [36]. As a tetraspanin, it contributes to the formation of specialized membrane microdomains that viruses often exploit to assemble and exit host cells [43,44]. For example, CD9 is enriched at sites of viral egress and can be incorporated into viral envelopes, influencing the efficiency and infectivity of particles such as HIV and other enveloped viruses [25,43]. Conversely, in certain contexts CD9 may act restrictively, limiting viral entry or fusion depending on the viral strain and host cell type [25]. These complex interactions highlight CD9 as both a potential facilitator and regulator of viral infection pathways [25].

Using complementary CRISPR/cas9 and siRNA strategies to knockdown endogenous expression of CD9 in Hela cells, we asked whether infectivity/spread of live EBOV/MARV as well as egress of live EBOV/MARV and VP40 VLPs was modulated compared to that in WT Hela cells. We found that expression of endogenous CD9 was indeed critical for efficient infectivity/spread of live EBOV and MARV, and equally critical for efficient egress of both VP40 VLPs and live EBOV and MARV in Hela cells. Consistent with these findings, CD9 depletion in Vero cells also impaired EBOV and MARV VLP release. These results identify CD9 as a previously unrecognized but important host determinant of filovirus egress.

Although the precise mechanism by which CD9 regulates filovirus egress remains to be determined, our BSL2 and BSL4 findings are consistent and correlate well in identifying host CD9 for the first time as a critical positive regulator of filovirus release. We speculate that CD9’s role in organizing microdomains and cytoskeletal interactions at the plasma membrane [45] is likely part of the mechanism by which it facilitates egress of EBOV and MARV. More detailed studies to further address the molecular mechanism by which CD9 promotes egress of EBOV and MARV and to study the potential role for filamin and other matrisome proteins as co-factors are currently ongoing.

CD9’s well-established roles in modulating ECM engagement, cell adhesion, and cytoskeletal dynamics [46,47,48] further underscore its potential importance. By organizing tetraspanin-enriched microdomains, CD9 influences cellular interactions with ECM components such as fibronectin, laminin, and collagen and regulates cytoskeletal dynamics, cell motility, and tissue remodelling [49,50,51,52,53]. Our preliminary data suggest that ECM stiffness may play a role in the plasma membrane-driven stages of filovirus entry and egress. Thus, we are currently investigating the dynamics of CD9–ECM interactions as a potential modulator of the filovirus lifecycle. More broadly, emerging ECM proteomics represents a promising avenue for the future discovery of how the ECM and ECM-associated proteins both dictate infectivity and respond to and adapt to pathogen infection and the disease state, potentially identifying ECM-associated targets for the development of new antiviral therapeutics.

In summary, our identification of CD9 as a positive regulator of EBOV and MARV egress highlights the importance of host membrane- and ECM-associated proteins in the filovirus lifecycle. Targeting such host dependencies offers a promising strategy for developing novel antivirals, and the discovery of CD9’s role provides a key entry point for defining conserved host pathways exploitable for broad-spectrum therapeutic development.

## Data Availability

The original contributions presented in this study are included in the article. Further inquiries can be directed to the corresponding author.
